# The association between short-term exposure to nitrogen dioxide and hospital admission for schizophrenia: A systematic review and meta-analysis

**DOI:** 10.1097/MD.0000000000035024

**Published:** 2023-09-29

**Authors:** Jiating Xu, Zhiyong Lan, Penghao Xu, Zhihua Zhang

**Affiliations:** a Department of General Psychiatry II, The Third Hospital of Quzhou, Quzhou City, China; b Department of Geriatric Psychiatry II, The Third Hospital of Quzhou, Quzhou City, China.

**Keywords:** meta-analysis, nitrogen dioxide, schizophrenia, systematic review

## Abstract

**Background:**

Ambient air pollution has been identified as a primary risk factor for mental disorders. In recent years, the relationship between exposure to ambient nitrogen dioxide (NO_2_) and the risk of hospital admissions (HAs) for schizophrenia has garnered increasing scientific interest, but evidence from epidemiological studies has been inconsistent. Therefore, a systematic review and meta-analysis were conducted to comprehensively identify potential correlations.

**Methods:**

A literature search in 3 international databases was conducted before December 31, 2022. Relative risk (RR) and corresponding 95% confidence intervals (CI) were calculated to evaluate the strength of the associations. Summary effect sizes were calculated using a random-effects model due to the expected heterogeneity (*I*^2^ over 50%).

**Results:**

A total of ten eligible studies were included in the meta-analysis, including 1,412,860 participants. The pooled analysis found that an increased risk of HAs for schizophrenia was associated with exposure to each increase of 10 μg/m^3^ in NO_2_ (RR = 1.029, 95% CI = 1.016–1.041, *P* < .001). However, the heterogeneity was high for the summary estimates, reducing the credibility of the evidence. In 2-pollutant models, results for NO_2_ increased by 0.3%, 0.2% and 2.3%, respectively, after adjusting for PM_2.5_, PM_10_ and SO_2_.

**Conclusions:**

This study provides evidence that NO_2_ exposure significantly increases the risk of hospital admission for schizophrenia. Future studies are required to clarify the potential biological mechanism between schizophrenia and NO_2_ exposure to provide a more definitive result.

## 1. Introduction

### 1.1. Social and economic burden of schizophrenia

Schizophrenia is identified as a severe mental disorder characterized by abnormalities in thought and cognition.^[1]^ It affects more than 24 million people worldwide and is among the top 15 leading causes of disability in the world.^[2,3]^ Empirical evidence shows that people with schizophrenia have impaired daily living skills and a higher risk of premature death than the general population.^[4]^ In addition to its impact on individuals, schizophrenia imposes a significant burden on society and the economy. People with schizophrenia, or those at risk of developing it, are more likely to engage in aggressive behavior towards others, potentially leading to violent crimes.^[5]^ Moreover, schizophrenia is one of the most socially and economically costly mental disorders. A comprehensive review of 56 studies indicated that the economic burden of schizophrenia ranged from 0.02% to 1.65% of GDP in different countries.^[6]^ Unfortunately, the prevalence of schizophrenia is on the rise in many countries, such as China and Korea.^[7,8]^ Many studies suggest that the primary contributors to schizophrenia include genetic, socioeconomic, environmental, and behavioral risk factors.^[4,9]^ The relationship between air pollution and schizophrenia has recently been highlighted.

### 1.2. Air pollution and mental illness: The potential association between NO_2_ and schizophrenia

Air pollution represents the most significant environmental health risk globally. According to the World Health Organization, over 90% of the world's population resides in areas where air pollution levels exceed the recommended limits.^[10]^ Exposure to particulate (PM_2.5_ and PM_10_) and gaseous pollutants (NO_2_, NO, SO_2_, O_3_, and CO) may trigger systemic and neuroinflammatory responses, possibly leading to psychopathology through altered neurotransmission and neurodegeneration.^[11–13]^ Therefore, it has been plausibly hypothesized that air pollution exposure may contribute to schizophrenia pathogenesis. Nitrogen dioxide (NO_2_) is a gaseous air pollutant produced by fossil fuel combustion in transportation. In studies that determine the association between gaseous pollutants and schizophrenia, NO_2_ has received relatively high attention from researchers, but the results remain controversial. Some studies reported a positive correlation between them,^[4,14]^ and others found no association.^[15,16]^ We, therefore, performed a systematic review and meta-analysis of original research articles to further clarify the potential associations between NO_2_ exposure and schizophrenia.

The effects of NO_2_ on schizophrenia can be classified into short-term and long-term effects, where the latter relates to cumulative exposure while the former pertains to an individual acute response. This study examines the association between short-term NO_2_ exposure and schizophrenia, with daily NO_2_ concentrations serving as the independent variable, and the risk of hospital admission for schizophrenia as the dependent variable. Furthermore, the results from the 2-pollutant models reported in the included studies were analyzed separately to control for the potential impact of other pollutants (such as PM_2.5_, PM_10_, SO_2_, etc) on the findings.

## 2. Materials & methods

### 2.1. Registration

The systematic review and meta-analysis have been registered and published in PROSPERO, with the assigned registration number CRD42022378942.

### 2.2. Ethics statement

In light of the fact that all analyses were conducted on previously published studies, and no patient consent or ethical approval was required.

### 2.3. Search strategy

This study was performed according to the preferred reporting items for systematic review and meta-analysis (PRISMA) statement (see Guidelines Checklist). The literature search of the PubMed, Embase and Web of Science electronic databases was independently conducted by 2 authors to identify any eligible epidemiological studies published up to December 31, 2022. A search strategy was detailed as following terms: (“nitrogen dioxide” OR “NO_2_” OR air pollutants) AND (“schizophrenia” OR “schizophrenics”) AND (“hospitalization” or “admission” or “admissions” or “visit” or “visits”). The reference lists of eligible studies and other related reviews were also screened to identify additional studies.

### 2.4. Study selection

Based on the predesigned PECO framework, the following eligibility criteria were selected in this study: (P) the study was performed among human participants; (E) the study focused on short-term exposure to NO_2_ (<30 days); (C) the study provided quantitative effect estimates with 95% CIs (or standard errors) by comparing humans exposed to lower air pollution levels with humans exposed to greater levels; and (O) schizophrenia confirmed by clinical assessment of the International Classification of Diseases (ICD). Studies that did not report the effect of short-term NO_2_ exposure on the risk of hospital admission for schizophrenia were excluded. Reviews, non-English studies, and animal experiments were also excluded.

**Figure 1.**
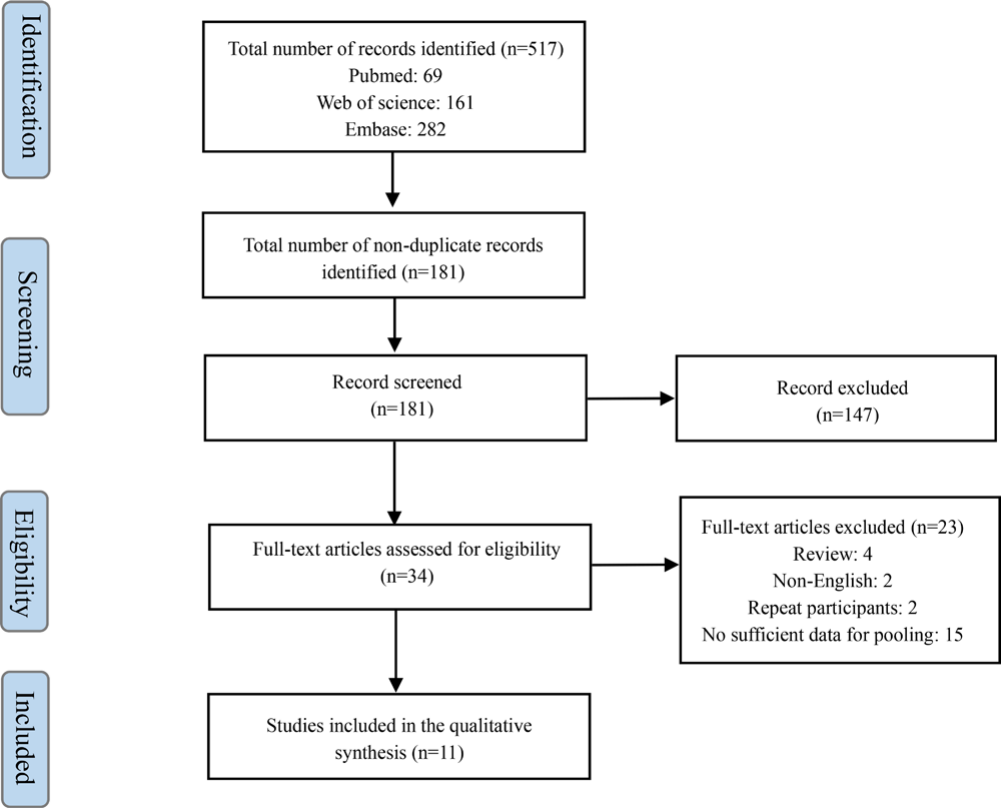
Flow chart of the study selection process.

After deduplication, 2 coauthors screened titles and abstracts for eligibility and retrieved the full texts of eligible studies for further assessment. Any disagreements in the process were resolved through group discussions until consensus was reached. The detailed process of eligible study selection is shown in Figure 1.

### 2.5. Data extraction and assessment of the methodological quality

Two coauthors independently extracted the following elements from eligible studies. They entered the data into a predesign Excel spreadsheet, including first-author name, publication year, country of origin, study design, study period, outcome definition, study participants, exposure assessment, adjustment variables, and main results. Any discrepancy was resolved by discussion with a third coauthor.

The study design of eligible studies included in this analysis included both time-series and case-crossover studies. To the best of our knowledge, there are currently no valid scales to assess the quality of these 2 types of studies. The scale suggested by Mustafic was used to assess their quality.^[17]^ The establishment of this quality assessment scale is based on previous successful experience, in particular the selection of several items from the New Castle Ottawa and Cochrane Risk of Bias Tools. This scale evaluates 3 items: validation of schizophrenia (absence of valid criteria was assigned 0, the presence of valid criteria was assigned 1), the quality of NO_2_ concentration measurements (0 was given if measurements were not conducted at least daily or with >25% missing data, and 1 if measurements were conducted at least daily or with <25% missing data), the quality of the adjustment for confounders (0 if no adjustment was made for long-term trends, season, and air temperature, 1 if only these 3 adjustments were made, 2 if an additional adjustment was made, either for relative humidity or day of the week and 3 if an adjustment was made for holidays in addition to those corresponding adjustments with a score of 2). A study with a score of 0 was categorized as “low quality,” and as “high quality” if it achieved a score of 5 (the highest score). All remaining study scores are classified as “medium quality.”

### 2.6. Standardization of data

To ensure comparability of outcomes across different studies, it was necessary to transform various types of effect sizes into relative risks (RR). As not all studies reported RR values, we converted the outcome of change rate of morbidity by dividing it by 100 and adding 1, thus obtaining the RR value. For regression coefficients, exponential transformation was used to derive the RR.^[18]^ Additionally, the extraction effect estimates per unit increase in NO_2_ exposure to a 10 μg/m^3^ increment were standardized prior to formal analysis. When studies reported NO_2_ concentration in ppb, the data were converted using 1 ppb NO_2_ = 46/22.4 μg/m^3^. Afterwards, RR for each study was standardized according to the following formula.^[18]^


$$\begin{array}{l} Standardised\;RR=exp\left[\begin{array}{c} \frac{natural\;Log\left(original\;RR\right)}{original\;increment}\;\times \;standardized\;increment \end{array}\right] \end{array}$$

In short-term exposure studies, schizophrenia admission risks with different lag patterns (single-day lags: lag 0, lag 1, lag 2…; cumulative lags: lag0–1, lag0–2…) may be reported. To facilitate pooling across studies, lags were selected according to the following criteria: if only 1 lag estimate was provided, the estimate was selected; or if multiple lags were provided, in order of preference, the lag that researchers focused on or preferentially stated was chosen, the lag that was statistically significant, or the lag with the largest effect estimates.^[19]^

### 2.7. Statistical analysis

Standardized RR values and 95% confidence intervals for each eligible study were selected for meta-analysis. Heterogeneity between studies was assessed using Cochran Q and *I*^2^ statistics. A *P* value of Cochran Q <0.1 was considered statistically significant for heterogeneity. The *I*^2^ statistic provides quantitative indicators of heterogeneity and inconsistency between studies. According to the experience of previous studies, *I*^2^ is equal to 0% to 25%, 25% to 50%, 50% to 75%, and 75% to 100% representing no heterogeneity, mild heterogeneity, severe heterogeneity, and extreme heterogeneity, respectively.^[20]^ Due to the considerable heterogeneity of this study, a random-effects model was chosen to pool effect sizes. A subgroup analysis was then conducted to explain the source of heterogeneity. Publication bias was visually assessed using a funnel plot for asymmetry and Egger test (*P* < .1 indicated the existence of publication bias). Additionally, the trim-and-fill method was used to test for contributions of publication bias to the overall effect. The meta-analyses were performed using STATA version 13.0 (Stata Corporation, TX).

## 3. Results

### 3.1. Literature retrieval and study characteristics

The search strategy identified 517 potentially relevant studies in 3 electronic databases. The full texts of 34 relevant articles were retrieved for further selection after removing duplicates, animal experiments, and reviews. Finally, eleven eligible studies were identified after excluding studies with non-short-term effects and did not report relevant outcomes (Fig. 1).

Table 1 details the baseline characteristics of the 11 studies in the meta-analysis.^[4,14,15,21–28]^ Seven of the 11 studies were undertaken in China (one included 3 sub-studies^[22]^), 2 in the USA, and 2 in South Korea. Most of these studies were performed in a time-series design (n = 9), and the remaining 2 used a case-crossover design, while the sample size of these studies ranged from 3218 to 649,052. All studies used data from national or regional ambient monitoring sites/centers for exposure measures. Three studies used chemiluminescence methods to measure NO_2_ concentration. Ten studies reported single-pollutant analysis (NO_2_) results, and 7 studies reported results from 2-pollutant models (NO_2_ plus other pollutants). For the diagnosis of schizophrenia, 7 and 3 studies used the ICD-10 and ICD-9 codes, respectively.

**Table 1:** Characteristics of the studies included in the meta-analysis.

Author, publication year (country)	Study design (study period)	Study participants	Female	Age	Outcome definition	Exposure data sources (assessment methods)	Adjustment variables	Main results (95% CI)	Quality assessment
Ji 2022 (China, Qingdao)^[15]^	Time series (2015–2019)	10,893	4103	5616 participants >45 years	ICD-10: F20-F20.9	Qingdao Environmental Monitoring Center (not reported)	Gender, age, and season, relative humidity, holiday, temperature and day of the week	NO_2_: Increment (10 μg/m^3^) lag0–6: RR = 1.044 (0.994–1.096) Lag5, +PM_2.5_: RR = 1..004 (0.989–1.020) Lag5, +PM_10_: RR = 1.006 (0.996–1.016) Lag0–7, +SO_2_: RR = 1..275 (1.116–1.457) Lag5, +CO: RR = 0..999 (0.998–1.001)	High
Li 2020 (China Huizhou)^[22]a^	Time series (2013–2018)	293,148	142,177	18–63 years: 253,866	ICD-10:F20-F29	Huizhou air monitoring stations (chemiluminescence methods)	Age, gender, temperature, relative humidity, holiday and day of the week	NO_2_: Increment (10 μg/m^3^) Lag0–3: RR = 1.041(1.023–1.058) Lag0, +PM_2.5_: RR = 1.051 (1.032, 1.070) Lag0, +PM_10_: RR = 1.050 (1.031, 1.071) lag3, +SO_2_: RR = 1.041 (1.023, 1.059	High
Li 2020 (China Shenzhen)^[22]b^	Time series (2016–2018)	649,052	351,137	18–63 years: 568,570	ICD-10:F20-F29	Shenzhen air monitoring stations (chemiluminescence methods)	Age, gender, temperature, relative humidity, holiday and day of the week	NO_2_: Increment (10 μg/m^3^) Lag0–3: RR = 1.083(1.065–1.101) Lag0, +PM_2.5_: RR = 1.092 (1.072, 1.112) Lag0, +PM_10_: RR = 1.089 (1.069, 1.109) Lag0, +SO_2_: RR = 1.088 (1.069, 1.108)	High
Li 2020 (China Zhaoqing)^[20]c^	Time series (2016–2018)	191,020	94,364	18–63 years: 153,580	ICD-10:F20-F29	Zhaoqing air monitoring stations (chemiluminescence methods)	Age, gender, temperature, relative humidity, holiday and day of the week	NO_2_: Increment (10 μg/m^3^) Lag0–3: RR = 1.018(0.998–1.038) Lag0, +PM_2.5_: RR = 1.014 (0.992, 1.037) Lag0, +PM_10_: RR = 1.013 (0.990, 1.036) Lag0, +SO_2_: RR = 1.016 (0.995, 1.038)	High
Bai 2018 (China Hefei)^[4]^	Time series (2014–2016)	11 373	5584	7984 participants >40 years	ICD-10:F20-F29	Hefei Environmental Monitoring Center (not reported)	Gender, age, and season, relative humidity, holiday, temperature, and day of the week	NO_2_: Increment (18 μg/m^3^) Lag0–1: RR = 1.10 (1.03–1.18) Lag0–1, +PM_2.5_: RR = 1.11(1.04, 1.18) Lag0–1, +CO: RR = 1.14 (1.06, 1.22)	High
Qiu 2022 (American)^[23]^	case–crossover study (2000–2016)	165,572	111,988	124,201 participants: 65–74 years	ICD-9 and ICD-10: 295.xx, F20.xx, F25.0x and F25.1x	US Environmental Protection Agency (not reported)	Age, race, gender, Medicaid eligibility, season, relative humidity, temperature, and day of the week	NO_2_: Increment (5ppb/10.25 μg/m^3^) Lag0–6: RR: 1.006 (1.002, 1.011)	Medium
Ruwan 2020 (American, California)^[14]^	Time series (2005–2013)	43,782	14,886	16,199 participants: 35–49 years	ICD-9-CM: codes 295, 297	U.S. Environmental Protection Agency, Air Quality System (not reported)	Age, race, gender, relative humidity, holiday, temperature and day of the week	NO_2_: Increment (10.79 ppb/22.12 μg/m^3^) Lag0: RR = 1..0045 (0.986, 1.0232)	High
Liang 2018 (China Xian)^[22]^	Time series (2010–2013)	34,865	15,990	20,919 participants: 21–40 years	ICD-10:F20	China National Environmental Monitoring Center (not reported)	Age, gender, season, relative humidity, temperature, and day of the week	NO_2_: Increment (10 μg/m^3^) Lag0: RR = 1.019 (1.010–1.028) Lag0, +PM_10_: 1.015 (1.003, 1.026) Lag0, +SO_2_: 1.010 (0.998, 1.023)	Medium
Duan 2018 (China, Tongling)^[23]^	Time series (2014–2016)	3469	1249	2446 participants ≥40 years	ICD-10 codes: F20–F29	China National Environmental Monitoring Center of Tongling City (not reported)	Gender, age, occupation, season, relative humidity, temperature, and day of the week	NO_2_: Increment (31 μg/m^3^) Lag0–3: RR: = 1.43 (1.18–1.73)	Medium
Lee 2022 (South Korea)^[26]^	case-crossover study (2008–2016)	15,917	9065	8910 participants ≥35 years	ICD-10 codes: F20, F22-F25, F28, and F29	Korea National Institute of Environmental Research (chemiluminescence methods)	Gender, age, season, influenza epidemics, holiday, temperature, relative humidity, barometric pressure, sunlight hours, rainfall, season, and day of the week	NO_2_: Increment (17.69ppb/36.26 μg/m^3^) Lag7: RR = 1.045 (1.013–1.078) Lag2, PM_2.5_: RR = 1.009 (0.976, 1.043)	Medium
Kim 2019 (South Korea)^[25]^	Time series (2014–2016)	67,561	33,276	41,653 participants >45 years	ICD-10 codes: F20–F29	Korea National Institute of Environmental Research (chemiluminescence methods)	Age, gender, temperature, relative humidity, air pressure, age, gender, day of the week, season, and patient addresses	NO_2_: Increment (10 μg/m^3^) Lag1: RR = 0.996 (0.965–1.028)	Medium
Chan 2018 (China Hongkong)^[25]^	Time series (2002–2011)	44,600	21,208	23,452 participants ≥60 years	ICD-9-CM: codes 295	Environmental Protection Department of Hong Kong (not reported)	Gender, age, temperature, day of study, day of year, day of week, relative humidity, season, and holiday	NO_2_: Increment (64 μg/m^3^) Lag0–8: RR = 1.70 (1.00–2.88)	High
Gao 2017 (China Beijing)^[28]^	Time series (2013–2015)	13,291	6925	36.8 ± 14.4 years	ICD-10 codes: F20–F29	Beijing Municipal Environmental Monitoring Center (not reported)	Gender, age, temperature, air pressure, sunshine hours, day of week, relative humidity, season, and holiday	NO_2_: Increment (10 μg/m^3^) Lag0–6, +PM_2.5_: RR = 1.011 (0.997, 1.025) Lag0–6, +PM_10_: RR = 1.018 (1.006, 1.030)	High

95% CI = 95% confidence interval, ICD = International Classification of Diseases, RR, relative risk.

### 3.2. NO_2_ exposure and the admission risk for schizophrenia

**Figure 2.**
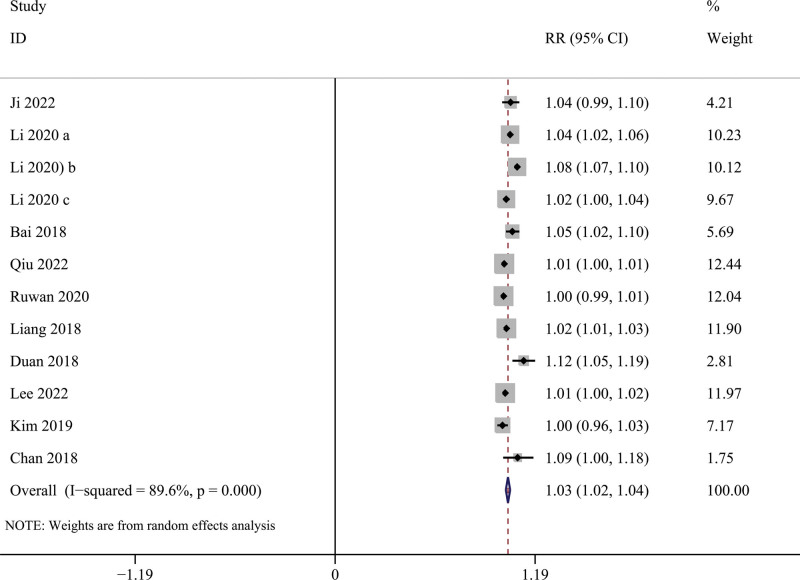
Forest plot of the associations between NO_2_ exposure and risk of hospital admission for schizophrenia. NO2 = nitrogen dioxide.

**Figure 3.**
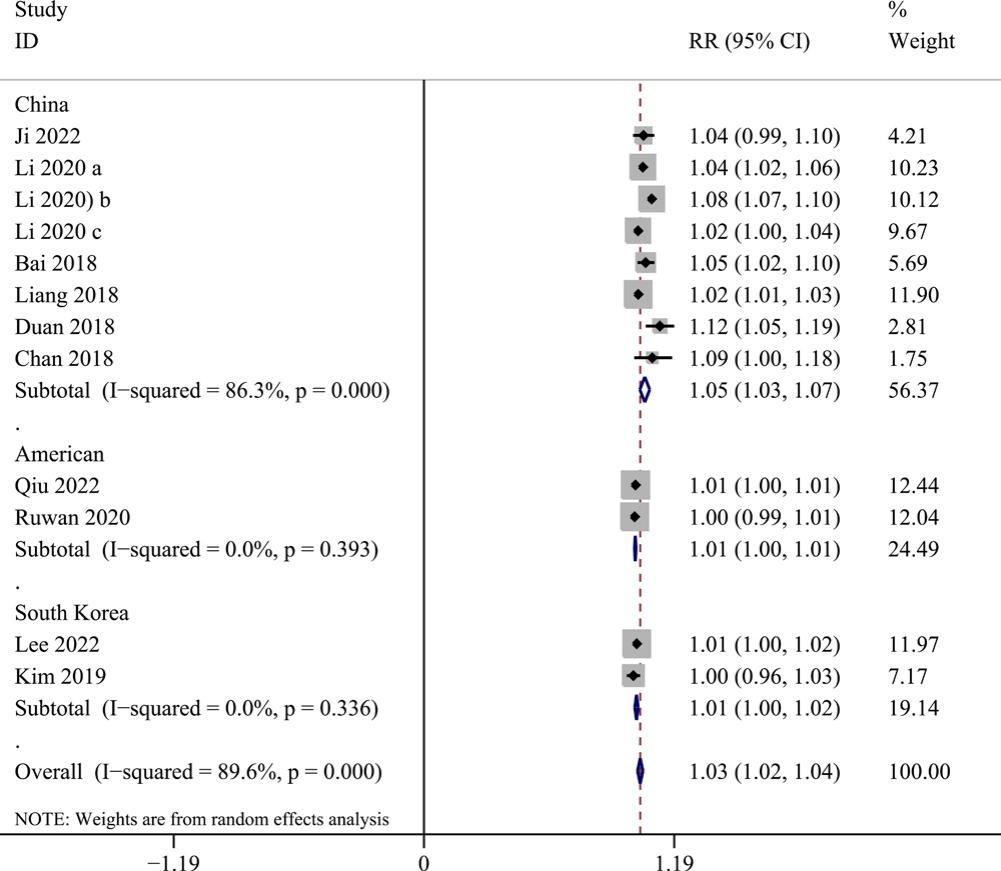
Results of a subgroup analysis of the association between NO_2_ exposure and risk of hospital admission for schizophrenia in different countries. NO_2_ = nitrogen dioxide.

Figure 2 shows the pooling effect estimates of the eligible studies that explored the associations of short-term NO_2_ exposure with hospital admissions for schizophrenia. Under the random-effects model, NO_2_ exposure is significantly associated with an increased hospital admission risk for schizophrenia (RR per 10 μg/m^3^ = 1.029, 95% CI = 1.016–1.041, *I*^2^ = 92.2%). In the subgroup analyses (Fig. 3), a significant association between NO_2_ exposure and the risk of hospital admission for schizophrenia was identified in these 3 countries. However, there is a discrepancy between their pooling effect sizes, the lowest in the USA is RR = 1.002, 95% CI = 1.001–1.003, *I*^2^ = 0%, South Korea is second with RR = 1.011, 95% CI = 1.003–1.019, *I*^2^ = 0 %, and the highest in China is RR = 1.050, 95% = 1.027–1.074, *I*^2^ = 86.3%. This result suggests that data from different countries may be a potential source of heterogeneity.

**Figure 4.**
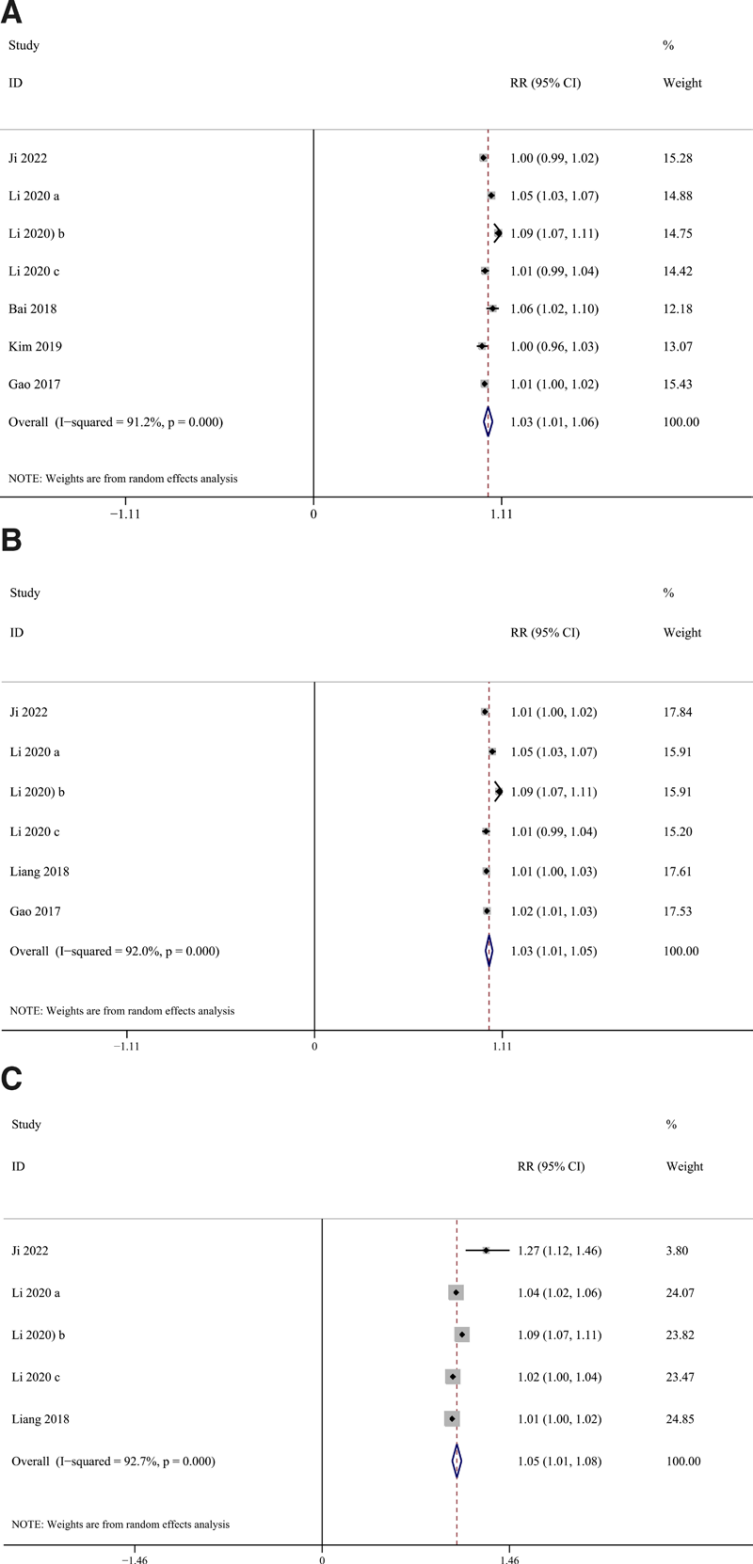
The analysis results of 2-pollutant models. (A) Adjusted PM_2.5_; (B) adjusted PM_10_; (C) adjusted SO_2_.

Figure 4 shows the results of a meta-analysis of the effect of 2 pollutants on hospital admissions (HAs) for schizophrenia. The impact of NO_2_ on schizophrenia was not substantially changed after adding PM_2.5_ (RR = 1.032, 95% CI = 1.006 to 1.058, *I*^2^ = 91.2%), PM_10_ (RR = 1.031, 95% CI = 1.010 to 1.052, *I*^2^ = 92.0%) and SO_2_ (RR = 1.048, 95% CI = 1.012 to 1.083, *I*^2^ = 92.7%).

### 3.3. Quality assessment and publication bias

All eligible studies were assessed for methodological quality. Six studies were of high quality, and 5 were of intermediate quality due to unadjusted holidays, influenza epidemics, etc. The quality assessment results and details are shown in Table 1 and Supplementary Table 1, http://links.lww.com/MD/J691 respectively.

**Figure 5.**
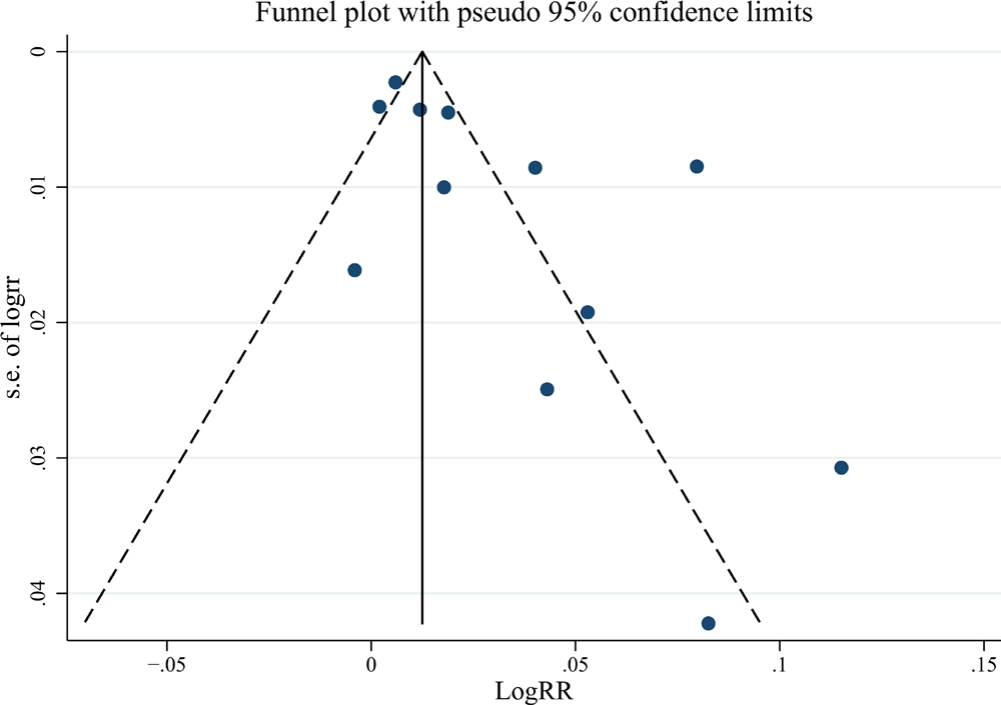
Funnel plot to evaluate potential publication bias.

Funnel plots and Egger test were applied to assess potential publication bias (Fig. 5). There is publication bias among the studies included (*P* = .006 <0.1). The trim-and-fill analysis identified 4 missing studies for the analysis, and the adjusted estimate was not substantially changed (RR = 1.025, 95% CI = 1.012–1.038).

## 4. Discussion

In the present systematic review and meta-analysis, ten studies from different countries were investigated to examine the correlation between short-term NO_2_ exposure and the risk of hospital admission for schizophrenia. Each 10 μg/m^3^ increase in NO_2_ concentrations is significantly associated with a 2.9% increased risk of hospital admission for schizophrenia. However, the results must be cautiously interpreted due to the high heterogeneity among the studies. By subgroup and trim-and-fill analyses, this correlation is unaffected by publication bias and heterogeneity. In a sense, this study provides synthesized evidence for previously inconsistent conclusions.

This study is the first comprehensive analysis to identify the association between schizophrenia and short-term exposure to NO_2_, providing essential evidence for the effects of NO_2_ exposure on mental disorders and the effect of environmental factors on schizophrenia. The results of the 2-pollutant model also confirm that the association was robust to co-pollutants (PM_2.5_, PM_10_, and SO_2_) adjustment. Similarly, several previous meta-analyses found short-term NO_2_ exposure to be an independent risk factor for depression among air pollutants.^[18,29]^ However, another previous meta-analysis reported that NO_2_ exposure was a protective factor for anxiety disorder.^[30]^ The results of these studies reveal that the effects of NO_2_ exposure on various mental disorders may differ. There was empirical evidence of the long-term effects of NO_2_ exposure on individuals developing schizophrenia. Two national cohort studies have demonstrated that higher concentrations of residential NO_2_ exposure during childhood were associated with an increased risk of later developing schizophrenia.^[10,31]^ The underlying pathophysiological mechanisms linking environmental pollutant exposure to schizophrenia remain unclear. However, Alan S. Brown et al have proposed the gene-environment interplay hypothesis to elucidate this association. This hypothesis builds upon the premise that genetic factors are the primary cause of schizophrenia and suggests that environmental factors increase an individual risk of developing schizophrenia through intermediate pathways such as oxidative stress, apoptosis, inflammation, and the HPA axis.^[32]^ Potentially, 3 pathways can be considered, gene-environment interactions, genes as antecedents of environmental exposure, and epigenetic effects.^[32]^

High heterogeneity is found among the ten studies included in this meta-analysis. After subgroup analysis, heterogeneity was primarily from the 6 studies within China. Studies in different countries also contributed a lower proportion of heterogeneity. Such heterogeneity is considered from the following perspective. The 6 studies from China (including 9 study cities), reports from Shenzhen (RR = 1.08), Hong Kong (RR = 1.08), and Tongling (RR = 1.12) had higher effect sizes than other cities. Firstly, socioeconomic factors between the different cities may contribute to this difference. In China, Shenzhen and Hong Kong are the main cities experiencing environmental stresses, including higher competitive pressures, more immigration, lower social cohesion and social support, and higher housing values, which have all been associated with developing schizophrenia.^[33,34]^ Secondly, Tongling economy is comprised primarily of copper mining and planting, which means many outdoor employees are exposed to air pollution for extended times, thereby increasing the risk of hospital admissions for schizophrenia. Thirdly, exposure level estimation errors vary due to differences in the density of ambient monitoring stations across studies (the higher the density, the more accurate the estimations), which may be an essential source of heterogeneity.^[35]^

Several limitations need to be acknowledged in this study. Firstly, data were obtained from only 3 countries, with the majority of studies (7/11) being conducted in China, which may restrict the generalizability of the findings. This limitation may be attributed to local contextual factors and publication bias, where studies with negative results are less likely to be prioritized for publication. It is noteworthy that China has been reported to have a higher prevalence of schizophrenia (2.64% globally^[36]^ compared to 65% to 8.3% in China^[7,37]^) and severe air pollution due to rapid industrialization. Secondly, although the methodological quality assessment scale employed in this study has been previously used in systematic reviews, its validity has not been scientifically established. Thirdly, publication bias may have resulted in an overestimation of the true effect size, as studies reporting non-significant effects may have remained unpublished. However, the trim-and-fill method did not reverse the initial outcomes. Fourthly, exposure assessment in all included studies was based on data from stationary ambient monitoring stations, which may have led to exposure misclassifications and information bias. Finally, differences in the intensity of air pollution exposure among individuals from different socioeconomic backgrounds within the same region may exist. Most of the included studies did not adjust for these factors, which may have influenced the study results.

### 4.1. Policy implications

This finding has significant policy implications for public health, as it highlights the need for preventive measures and interventions to reduce the incidence of this debilitating mental illness. Policy-makers should consider implementing targeted interventions aimed at reducing air pollution levels in areas with high population density or high levels of industrial activity. These interventions may include stricter regulations on industrial emissions, as well as the promotion of clean technologies and renewable energy sources, such as carbon neutrality policy.^[38,39]^ Additionally, public health campaigns can raise awareness about the adverse effects of air pollution on mental health, encouraging individuals to take measures to reduce their exposure to pollutants. In terms of healthcare policy, our findings underscore the importance of early detection and treatment of schizophrenia. Policy-makers should prioritize the development of accessible and effective mental health services, including screening programs, early intervention programs, and evidence-based treatment options.

## 5. Conclusions

In summary, this systematic review and meta-analysis suggests an association between short-term exposure to NO_2_ and HAs for schizophrenia. However, given the limitations identified in the study, further research is necessary, particularly from countries with high levels of ambient air pollution, such as Africa and India, to clarify the plausibility of this association. Moreover, it is recommended that future studies adjust for potential confounding factors, such as holidays, meteorological factors, and flu epidemics, to provide insight that could benefit relevant public and health policies.

## Author contributions

**Conceptualization:** Jiating Xu, Zhihua Zhang.

**Data curation:** Zhiyong Lan, Penghao Xu.

**Formal analysis:** Jiating Xu, Zhiyong Lan, Penghao Xu.

**Methodology:** Zhiyong Lan, Penghao Xu, Zhihua Zhang.

**Visualization:** Zhiyong Lan.

**Writing – original draft:** Jiating Xu.

**Writing – review & editing:** Penghao Xu, Zhihua Zhang.

## Supplementary Material


