# Supplementary material for: The association between short-term exposure to nitrogen dioxide and hospital admission for schizophrenia: A systematic review and meta-analysis
Source: Medicine (Baltimore). 2023 Sep 29;102(39):e35024. doi: 10.1097/MD.0000000000035024 (PMC10545286; doi:10.1097/MD.0000000000035024)
Supplement: Supplementary file 1 [file medi-102-e35024-s001.docx]

Supplementary table 1. Details of the quality assessment of the included studies.

| Author, publication year (country) | Validation of schizophrenia | Measurements quality of NO_2_ concentration | Quality of the adjustment for confounders | | |
| --- | --- | --- | --- | --- | --- |
|  |  |  | Long-term trends, season, and air temperature | Relative humidity or day of the week | Holidays or influenza |
| Ji 2022 (China, Qingdao) | 1 | 1 | 1 | 1 | 1 |
| Li 2020 (China Huizhou) a | 1 | 1 | 1 | 1 | 1 |
| Li 2020 (China Shenzhen) b | 1 | 1 | 1 | 1 | 1 |
| Li 2020 (China Zhaoqing) c | 1 | 1 | 1 | 1 | 1 |
| Bai 2018 (China Hefei) | 1 | 1 | 1 | 1 | 1 |
| Qiu 2022 (American) | 1 | 1 | 1 | 1 | 0 |
| Ruwan 2020 (American, California) | 1 | 1 | 1 | 1 | 1 |
| Liang 2018 (China Xian) | 1 | 1 | 1 | 1 | 0 |
| Duan 2018 (China, Tongling) | 1 | 1 | 1 | 1 | 0 |
| Lee 2022 (South Korea) | 1 | 1 | 1 | 1 | 0 |
| Kim 2019 (South Korea) | 1 | 1 | 1 | 1 | 0 |
| Chan 2018 (China Hongkong) | 1 | 1 | 1 | 1 | 1 |
| Gao 2017 (China Beijing) | 1 | 1 | 1 | 1 | 1 |
